# Andersen–Tawil Syndrome With Novel Mutation in *KCNJ2*: Case Report

**DOI:** 10.3389/fped.2021.790075

**Published:** 2022-01-31

**Authors:** Jisook Yim, Kyoung Bo Kim, Minsun Kim, Gun Dong Lee, Myungshin Kim

**Affiliations:** ^1^Department of Laboratory Medicine, College of Medicine, The Catholic University of Korea, Seoul, South Korea; ^2^Catholic Genetic Laboratory Center, Seoul St. Mary's Hospital, College of Medicine, The Catholic University of Korea, Seoul, South Korea; ^3^Department of Laboratory Medicine, Keimyung University School of Medicine, Daegu, South Korea

**Keywords:** *KCNJ2* gene, KIR2.1, periodic paralysis, long QT syndrome, potassium channel, genetic disorder, dysmorphic feature, Andersen–Tawil syndrome

## Abstract

Andersen–Tawil syndrome (ATS) is a rare autosomal dominant disorder characterized by a classic symptom triad: periodic paralysis, ventricular arrhythmias associated with prolonged QT interval, and dysmorphic skeletal and facial features. Pathogenic variants of the inwardly rectifying potassium channel subfamily J member 2 (KCNJ2) gene have been linked to the ATS. Herein, we report a novel *KCNJ2* causative variant in a proband and her father showing different ATS-associated symptoms. A 15-year-old girl was referred because of episodic weakness and periodic paralysis in both legs for 2–3 months. The symptoms occurred either when she was tired or after strenuous exercise. These attacks made walking or climbing stairs difficult and lasted from one to several days. She had a short stature (142 cm, <3rd percentile) and weighed 40 kg. The proband also showed orbital hypertelorism, dental crowding, mandibular hypoplasia, fifth-digit clinodactyly, and small hands. Scoliosis in the thoracolumbar region was detected by chest X-ray. Since she was 7 years old, she had been treated for arrhythmia-associated long QT interval and underwent periodic echocardiography. Brain MRI revealed cerebrovascular abnormalities indicating absence or hypoplasia of bilateral internal carotid arteries, and compensation of other collateral vessels was observed. There were no specific findings related to intellectual development. The proband's father also had a history of periodic paralysis similar to the proband. He did not show any cardiac symptoms. Interestingly, he was diagnosed with hyperthyroidism during an evaluation for paralytic symptoms. Clinical exome sequencing revealed a novel heterozygous missense variant: Chr17(GRCh37):g.68171593A>T, NM_000891.2:c.413A>T, p.(Glu138Val) in *KCNJ2* in the proband and the proband's father.

## Introduction

Andersen–Tawil syndrome (ATS; MIM 170390) is a rare autosomal dominant disorder characterized by a classic triad of recurrent flaccid muscle weakness (periodic paralysis), cardiac arrhythmias, and distinctive skeletal and facial features (1). Since the first ATS case presenting with muscle weakness, extrasystoles, and multiple developmental anomalies was reported (2), ATS prevalence is estimated at 0.8–1 in 1,000,000 (3, 4). However, the actual prevalence is expected to be higher than previously reported because the reported prevalence is from a specific population and subgroup of ATS patients (5) and ATS is a rare disease with a broad phenotypic heterogeneity that is difficult to diagnose. Affected individuals present with episodes of periodic paralysis or cardiac symptoms in their first or second decade (6); however, highly variable phenotypes and incomplete penetrance make diagnosis challenging. Cardiac arrhythmias include ventricular arrhythmias associated with prolonged QT interval and prominent U waves. Skeletal and facial features include low-set ears, broad forehead, ocular hypertelorism, small mandible, dental abnormalities, fifth digit clinodactyly, syndactyly, short stature, and scoliosis (7). The *KCNJ2* and *KCNJ5* are causative genes of ATS. *KCNJ2* on chromosome 17q24.3 encodes an inward rectifier potassium channel protein (Kir2.1), which plays an important role in setting and stabilizing the resting membrane potential primarily in the skeletal muscles, heart, and brain (8). *KCNJ5* (11q24.3) encodes a G-protein-activated inward rectifier potassium channel 4 protein (Kir3.4) that induces an inhibitory effect on the inward rectifier potassium current (9). Therefore, the diagnosis of ATS is established in individuals with two or more symptoms of the classic triad or individuals with one of the classic triad and at least one family member with ATS and/or identification of pathogenic variants in *KCNJ2* and *KCNJ5*. Here, we report a family with ATS with a novel causative missense variant in *KCNJ2* c.413A>T, p.(Glu138Val) and compare their phenotypes along with a literature review.

## Case Report

The proband is a 15-year-old girl who presented with recurrent episodes of leg weakness and paralysis lasting several days beginning 2–3 months prior. These episodes recently became more severe and frequent, occurring at least twice a week. The severity of the weakness during the episodes varied from mild (weakness and difficulty climbing stairs) to severe (inability to walk). Triggers were intensive exercise or feeling tired. Clinical evaluation included medical and family history and previous medical record review where applicable. She had been taking flecainide for an arrhythmia associated with a long QT interval since she was 7 years old and had been receiving regular echocardiogram (ECG) follow-up. Her serum potassium levels tested during hospitalization were normal. Thyroid function test results were also within the reference interval. Electromyography (EMG) showed no specific findings. Brain magnetic resonance imaging (MRI) revealed suspicious congenital absence or hypoplasia of both internal carotid arteries (ICA) with supply to the anterior circulation via carotid-vertebrobasilar anastomoses and hypertrophy of the posterior communicating artery (PCOM). No intellectual disability was observed during development. Chest X-rays showed mild scoliosis in the thoracolumbar region. During physical examination, she was 142 cm tall (<3rd percentile) and weighed 40 kg (3rd percentile). Additional physical examination during genetic counseling revealed that the proband had dysmorphic features, including orbital hypertelorism, mandibular hypoplasia, dental crowding, clinodactyly of the fifth finger, and small hands.

Family history revealed that her 37-year-old father had similar episodes that began during adolescence. He was hospitalized for three quadriplegia attacks, and he took potassium supplement 600 mg once or twice a year when he experienced generalized weakness attacks. His potassium levels were low (lowest: 2.2 mmol/L) during his acute episodes. His medical history included transient hyperthyroidism but no cardiac symptoms. Since age 25, he had not experienced a paralysis severe enough to be hospitalized but continued to have periodic paralysis once every few years. His paralysis events were mild and improved without going to the hospital by taking a potassium supplement, applying hot compresses, and receiving massages. However, from March to May 2020, at the age of 35, he was hospitalized almost every 2 weeks with severe general paralysis during which he could not even move his fingers. His blood thyroid function test indicated hyperthyroidism. After taking thyroid medication for 2–3 months, he underwent three thyroid function tests, all of which were within the normal range. He remained well without any uncomfortable symptoms after that. However, detailed records (i.e., thyroid hormone levels) were not obtained as data by the hospital at that time. His height and weight were 163.5 cm (≈3rd percentile) and 51.25 kg, respectively. Hypertelorism, small mandible, tooth crowding, and small hands were also observed. Table 1 provides a summary of the clinical characteristics of our patients.

**Table 1:** Clinical details of ATS patients with pathogenic/likely pathogenic variants in Kir2.1 at position 138.

**Patients**	**Causative variant *KCNJ2* in amino acid 138**	**Sex/age at diagnosis (years)**	**Age with first symptoms (years)**	**Typical triad of Andersen–Tawil syndrome**			**Other additional features**
				**Periodic paralysis**	**Cardiac arrhythmia**	**Dysmorphic features**	
Proband	E138V	F/15	7	Yes	Yes (long QT interval)	Short stature (<3 percentile), hypertelorism, micrognathia, dental crowding, clinodactyly of the fiftth finger, small hands	Cerebrovascular abnormality (congenital hypoplasia of both internal carotid arteries)
Proband's father	E138V	M/37	19	Yes	No	Short stature (163.5 cm), hypertelorism, micrognathia, dental crowding, small hands	Transient hyperthyroidism and ictal exacerbation of periodic paralysis
Kostera-Pruszczyk et al. (10), same position variant case as our patients	E138K	M/5	*In utero*	Yes	Yes (long QT interval)	Short stature (<3 percentile), hypertelorism, micrognathia, clinodactyly of the fifth toe, syndactyly of the second and third toes	Not described

## Genetic Testing

**Figure 1:**
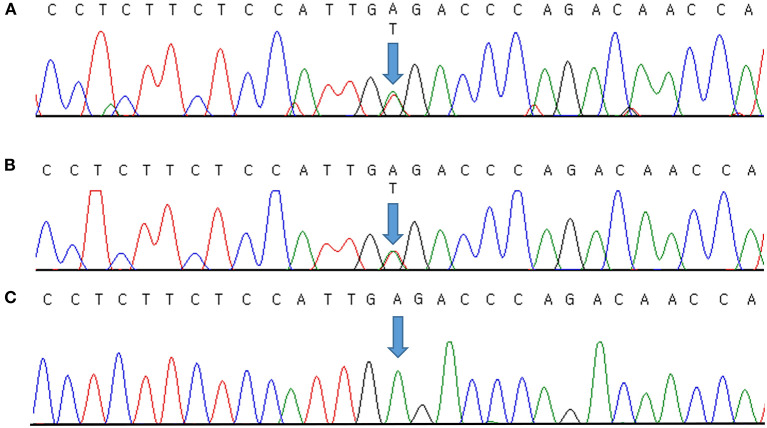
Molecular characterization of a family with Andersen–Tawil syndrome. Sequence chromatogram showing the position of the c.413A>T transversion (NM_000891.2) that led to the p.(Glu138Val) missense variant. The variant was heterozygous in the proband **(A)** and her father **(B)**. Sanger sequencing result of an unaffected control **(C)**. Variants are annotated according to The Human Genome Variation Society nomenclature.

Genomic DNA was extracted from peripheral blood samples using the QIAsymphony DSP DNA mini kit (Qiagen, Hilden, Germany). Sequencing was performed using a TruSight One Expanded sequencing panel (Illumina Inc., San Diego, CA, USA) consisting of 6,700 genes associated with known Mendelian genetic disorders on a NextSeq 550 (Illumina, CA, USA). The obtained sequence reads were aligned to the human reference genome sequence 19 (hg19) from University of California, Santa Cruz (UCSC) [original GRCh37 from National Center for Biotechnology Information (NCBI), February 2009] using Burrows–Wheeler Aligner (BWA version 0.7.7-isis-1.0.0). The BAM files were then processed by base quality recalibration, duplicate removal, and local realignment following the Genome Analysis Toolkit (GATK version 1.6-23-gf0210b3) for variant calling. Annotation and filtering were performed with Illumina Annotation Service (IAS version 3). Clinical exome sequencing revealed a novel causative heterozygous missense Chr17(GRCh37):g.68171593A>T, NM_000891.2:c.413A>T, p.(Glu138Val) variant in *KCNJ2*, which was thought to be causal of ATS syndrome. This domain (H5, intramembrane pore loop) is highly preserved, and the mutation is predicted to be damaging by the protein function prediction software SIFT and PolyPhen-2. It was not present in the general population databases such as the Genome Aggregation Database (gnomAD; https://gnomad.broadinstitute.org/), the Single Nucleotide Polymorphism Database (dbSNP; https://www.ncbi.nlm.nih.gov/snp/), ClinVar (http://www.ncbi.nlm.nih.gov/clinvar/), Human Gene Mutation Database (HGMD; http://www.hgmd.cf.ac.uk/ac/index.php), a public archive of the relationship between human variants and phenotypes, and the Korean Reference Genome Database (KRGDB; http://coda.nih.go.kr/coda/KRGDB/index.jsp) as an ethnic-specific Korean population database. We could not find the variant in our case in any previous reports. The identified variants were assessed according to the standards and guidelines for the interpretation of sequence variants in Mendelian disorders of the American College of Medical Genetics and Genomics and the Association for Molecular Pathology (ACMG/AMP) (11), with PM2 (absent from population database), PM5 (novel missense change at an amino acid residue where a different missense change determined to be pathogenic has been seen before), PP2 (missense variants are a common mechanism of disease), PP3 (pathogenic computational evidence), and PP4 (phenotype specific for gene) relevant evidence. Therefore, we classified the variant as likely pathogenic. It was validated by Sanger sequencing (Figure 1A) and then confirmed diagnosed as ATS. Because a causative variant was identified in the proband, Sanger sequencing of the causative variant was also performed on her symptomatic father, and the same variant that was consistent with his history of periodic paralysis were identified (Figure 1B). Figure 1C shows the Sanger sequencing result of an unaffected control on this position. No other pathogenic variants in genes associated with other periodic paralysis disorders (*CACNA1S, SCN4A*, or *KCNJ18*) were identified in the proband. The father's parents and two brothers were asymptomatic until now. Although the proband's 11-year-old brother and 4-year-old sister had no symptoms, genetic testing was recommended to all at-risk family members during genetic counseling.

## Discussion

In this case report, we identified two ATS patients in a family with the novel *KCNJ2* c.413A>T variant. Our proband had all triad symptoms, while her father had only two symptoms and no cardiac symptoms. It is very challenging to diagnose ATS early due to the rarity and high degree of phenotypic variability. Fifty-eight to seventy-eight percent of ATS patients present with all three symptoms (12). Moreover, patients can present with only one of the triad symptoms (5). Dysmorphic features are usually subtle in ATS patients (13), as shown in our patients (we recognized retrospectively), and are easy to overlook unless suspected of ATS. Therefore, ATS should always be considered even in patients with only periodic paralysis or cardiac arrhythmias.

Approximately 60% of ATS cases have causal variants in *KCNJ2* (6, 14). Most *KCNJ2* pathogenic variants in ATS are missense variants (1, 6) that result in loss of function of the Kir2.1 channel and is expressed in skeletal muscle, heart, and bone. This channel plays a role in prolonged depolarization of the action potential, and thereby cause periodic paralysis and cardiac arrhythmia (14). Additionally, *KCNJ2* variants can affect skeletal dysmorphic features because *KCNJ2* is expressed during the early stages of craniofacial development in *Xenopus* and mice (15). However, as reports by variable penetrance of ATS cases suggest, a subset of individuals with pathogenic variants do not exhibit any associated symptoms (16–18). A subset of *KCNJ2* pathogenic variants that cause ATS is identified at the phosphatidylinositol-4,5-biphosphate (PIP2) binding site, a known regulator of the Kir2.1 channel. Another subset of *KCNJ2* pathogenic variants is found in the pore-forming loop of Kir2.1. In addition, pathogenic variants in regions such as slide helix have also been reported (5, 19–21), suggesting various mechanisms. The p.(Glu138Val) variant in our patients was located in the pore region of the Kir2.1 channel. Another study showed the same position but other missense change (c.412G>A, p.E138K) as our case variant (c.413A>T, p.E138V) (10). Although not able to perform physiological studies to identify the nature of the alteration in channel function, other studies showed that this subunit pathogenic variant was unable to form functional homomultimeric channels, abolishing channel function (10, 18). And Yang et al. also described that position 138, a negatively charged residue in the pore loop of Kir2.1, had a strong effect on the stability of the pore loop (22, 23). Additional *in silico* analyses predicted deleterious (SIFT score: 0.05), probably damaging (polyphen-2 score: 1.000), pathogenic (REVEL score: 0.972), and disease-causing (MutationTaster) effects; thus, this variant may cause complete loss of channel function.

We found additional features in our patients. The proband had congenital hypoplasia of the ICA, identified by brain MRI. Although we did not find any reports that also reported vascular abnormalities in ATS, central nervous system involvement including mild learning disability was frequently observed in ATS patients. Because potassium channels contribute to angiogenesis in cancer (24), it is worth investigating a possible association between cerebrovascular development and *KCNJ2* variant. Meanwhile, the proband's father had a history of transient hyperthyroidism with exacerbated periodic paralysis that was consistent with previous studies' findings. They demonstrated that the paralysis symptoms were aggravated in patients with ATS secondary to thyrotoxicosis or co-existence of both disorders (25, 26). The heterogeneous phenotypes between the proband and her father demonstrated the weak genotype–phenotype correlation in ATS. This is further evidenced by a previously reported case with the missense variant of same position 138 who had earlier onset of more severe periodic paralysis (1 year old) and arrhythmia (*in utero*) than our proband (7 years old).

In summary, we identified a novel causative variant in *KCNJ2* in ATS patients and provided detailed clinical findings to expand the genotype–phenotype correlation. The heterogeneous phenotype of ATS should alert physicians to perform genetic analysis for ATS even in patients with only one or two among the typical triad symptoms.

## Data Availability Statement

The raw data supporting the conclusions of this article will be made available by the authors, without undue reservation.

## Ethics Statement

The studies involving human participants were reviewed and approved by Seoul St. Mary's Hospital Institutional Review Board. Written informed consent for participation was not provided by the participants' legal guardians/next of kin: Because the patient did not return to our hospital, written consent could not be obtained. However, it has been approved by the Ethics Committee of our hospital, and the patient's identification information is not included in this case.

## Author Contributions

JY and MyK wrote the manuscript. JY, KK, MiK, and GL conducted genetic analyses. All authors contributed to the manuscript.

## Conflict of Interest

The authors declare that the research was conducted in the absence of any commercial or financial relationships that could be construed as a potential conflict of interest.

## Publisher's Note

All claims expressed in this article are solely those of the authors and do not necessarily represent those of their affiliated organizations, or those of the publisher, the editors and the reviewers. Any product that may be evaluated in this article, or claim that may be made by its manufacturer, is not guaranteed or endorsed by the publisher.
